# Drug-Induced Liver Injury with Novel Fat Burner AlbutarexV2 in an Active Duty Sailor

**DOI:** 10.1093/milmed/usaf175

**Published:** 2025-05-08

**Authors:** Lt Jessica Lilley, Melanie Wiseman, Brett Sadowski

**Affiliations:** Internal Medicine Residency, Naval Medical Center San Diego, San Diego, CA 92134, USA; Division of Gastroenterology, Department of Medicine, Naval Medical Center San Diego, San Diego, CA 92134, USA; Division of Gastroenterology, Department of Medicine, Naval Medical Center San Diego, San Diego, CA 92134, USA

## Abstract

Drug-induced liver injury ranges from asymptomatic to liver failure with supplements becoming an increasingly recognized cause. This case highlights the unique supplement, AlbutarexV2, and the severe cholestatic liver injury it induced with resolution occurring in six months. With steadily increasing obesity rates and supplement use, weight-loss supplements are gaining popularity with various claims of increasing energy expenditure or metabolic rate. Recognition of adverse effects from these therapies is critical to aid in identification and cessation of the agent and helping ban harmful supplements. This case demonstrates the danger of “fat-burners” and delineates the protracted and prolonged liver injury of AlbutarexV2.

## INTRODUCTION

Drug-induced liver injury (DILI) ranges from asymptomatic transaminitis to acute liver failure and is classified by clinical presentation, mechanism of hepatotoxicity, and histological appearance of liver biopsy.^1^ For the clinical presentation, DILI is defined by elevation in liver chemistries, but it can range from a hepatocellular pattern to a cholestatic pattern.^1^ Hepatocellular injury has 3 times the upper limit of normal (ULN) elevation of transaminases, whereas in cholestatic injury alkaline phosphatase (ALP) is three times the ULN.^1^ Mixed pattern of injury has elevations in both transaminases and ALP.^1^ The diagnostic evaluation includes duration of exposure, symptoms present, sufficient exclusionary tests (viral serology, imaging, autoimmune work-up), and liver biopsy if necessary.^2^ Because of underreporting and comprehensive diagnostic criteria, DILI incidence is difficult to truly ascertain, but the incidence has been reported between 13.9 and 19.1 cases per 100,000 people per year.^3^

Multiple risk factors have been implicated for development of DILI including female sex, higher drug doses, longer duration of use, and older age.^3^ The higher incidence in women is theorized to be due to hormonal interactions while the risk factor of older age is thought to be because of increased prescription drug use allowing for more interactions to result in adverse events.^3^ The U.S. Dietary Supplement Health and Education Act outlined the legal definition and labeling for dietary supplements and placed supplements under the regulation of the Food and Drug Administration (FDA).^4^ Although the FDA has issued formal guidance on “Good Manufacturing Practice” to attempt to ensure proper preparation and labeling of supplements, the sheer volume of supplements produced leads to many of these supplements and their unsupported health benefit claims never receiving any oversight or regulation.^4^ Supplements are not required to provide information on the bioactivity or provide their specific formulations instead listing them as “proprietary blends.”^4^

## CASE REPORT

An overweight 22-year-old male active duty sailor presented with 7 days of painless jaundice and pruritus found to have a severe mixed and predominantly cholestatic liver injury in the setting of fat burner supplementation use (**Table 1**). One week before the development of symptoms, the patient started Albutarex V2 to meet required weight standards for the military and continued usage through the jaundice and pruritus (**Table 2**). The patient had not received antimicrobials or any other prescription drugs associated with DILI before his initial presentation. After discontinuing the supplement, the patient’s labs peaked 3 months after with AST 70 (Reference: 12-39), ALT 65 (Reference: 17-63), ALP 326 (Reference: 40-129), and bilirubin 41.66 (Reference: 0.15-1) with an R factor 0.6. Full resolution of the patient’s liver chemistries occurred at 6 months after supplementation cessation (**Table 1**). A broad work-up for alternative causes of liver injury including viral hepatitis, autoimmune hepatitis, primary biliary cholangitis (PBC), Wilson’s disease, syphilis, and HIV was negative (**Table 3**). Magnetic resonance cholangiopancreatography (MRCP) showed normal hepatic morphology with periportal edema and no evidence of any infiltrative process and normal bile ducts. While the patient had a positive antinuclear antibody (ANA), he also had a negative antismooth muscle antibody and total immunoglobulin G. It would also be atypical for autoimmune hepatitis to have profound elevation in bilirubin and a mild aminotransferase elevation pattern. Given the patient’s positive AMA, he was empirically trialed on Ursodiol to relieve his intractable pruritus. However, based on the patient’s demographic and unremarkable MRCP making PBC unlikely, Ursodiol was discontinued when it did not alleviate the patient’s pruritus. Given the progressive liver injury, a liver biopsy was obtained and interpreted by a pathologist, which revealed severe bland canalicular cholestasis without evidence of autoimmune hepatitis nor infiltrative process, consistent with DILI (Supplemental Figure 1).

**Table 1.** Trends in Laboratory Data Over Time.

	Day 0	Day 1	Day 2	Day 10	Day 23	Day 25	Day 104
AST, U/L (Reference: 12–39)	55	55	55	66	70	54	33
ALT, U/L (Reference: 17–63)	137	110	101	76	65	53	42
ALP, U/L (Reference: 40–129)	186	170	178	243	326	254	71
Bilirubin, Total, mg/dL (Reference: 0.15–1)	17.24	15.47	17.86	34.68	41.66	36.94	0.87
Bilirubin, Direct mg/dL (Reference: 0.2–0.3)	12.0	-	12.3	29	41.5	24.9	Not obtained
PT, s (Reference: 12–15)	12.8	13.4	13.4	13.9	16.6	16.7	13.6
INR (Reference: 0.8–1.2)	1.0	1.0	1.0	1.1	1.3	1.3	1.0
Eosinophils, % (Reference: 0–6)	2.2	3.8	3.8	3.8		2.9	2.1

**Table 2.** Table of ingredients from Fat Burner AlbutarexV2.

Energy	0 kj
Protein	0 g
Fat, Total	0 g
Carbohydrate Total	0 g
Sugar	0 g
Sodium	0 mg
Albutarex V2 Super Shred Complex	Caffeine (300 mg), Huperzine A 1%, Piperine, Taurine, N-Acetyl-L-Tyrosine, Theobromine, Theanine, Hordenine HCL, LeanGBB, 6-Paradol, Acetyl-l-Carnitine, African Mango (Seed) 10:1 Extract, Garcinia, Cambogia Extract, Coleus Forskolin (Root) Std to 20% Forskolin
Other ingredients	Contains Maltodextrin, Citric Acid, Natural and Artificial Flavors, Sucralose, FD&C Red #40

**Table 3.** Additional Work-up Performed for Acute Liver Injury.

Hep A IgM antibody	Nonreactive
Hep A antibody	Reactive
HBsAg	Nonreactive
Hep B surface antibody	Reactive
Hep B core antibody	Nonreactive
IgM anti-HBc	Nonreactive
Hep C antibody	Nonreactive
HCV RNA	Note detected
Hep D virus	Negative
Hep E virus	Negative
EBV IgM, U/mL	< 36
EBV DNA quantitative PCR	Negative
CMV Ab IgM, U/mL	<30.0
CMV PCR	Negative
ANA	Positive, 1:80
Smooth muscle antibody (Units)	8
Ceruloplasmin, mg/dL	27
Acetaminophen level, mg/dL	<5
Salicylate level, mg/dL	<0.3
HIV 1/2 AG/AB	Negative
Rapid plasma reagin	Non-reactive
IgG subclass, 4 mg/dL (Reference: 2.4–121)	110
IgG total mg/dL (Reference: 600–1,600)	1,181
Mitochondrial antibody units	37.1 and 27.2 on repeat

## DISCUSSION

We report the case of a 22-year-old male active duty sailor who developed a severe, predominately cholestatic liver injury following the use of a fat burner supplement to meet military weight standards. Using the Naranjo Adverse Drug Reaction Probability Scale, this case has a score of 7 indicating that the presentation is probably consistent with a drug reaction. This highlights the concerns of the usage of similar supplements for weight loss as independent to age, geographic location, ethnicity, or socioeconomic status, obesity rates are increasing exponentially with nearly one-third of the world population being classified as obese or overweight.^5^ While the global rise in obesity is a complex health issue with multifaceted causes including sedentary lifestyles, diets, and genetic factors, it poses a unique and significant public health challenge.^6^ Not only does obesity increase the risk for chronic diseases, the new quick-fix from weight-loss supplement use has begun to directly impact health care with supplement-associated injuries. During 2017-2018, 57.6% of adults in the United States had used a dietary supplement in the past month with prevalence increasing with age and among women.^7^ With steadily increasing obesity rates and exponentially increasing dietary supplement use, loosely regulated therapies aimed at weight loss are more commonly utilized. Weight loss supplements or “fat burners” are typically marketed with claims of increasing energy expenditure by increasing the resting metabolic rate to lead to weight loss.^8^ According to the National Institute of Health Office of Dietary Supplements, 15% of U.S. adults have used a weight-loss dietary supplement at some point with an annual revenue of almost $2.1 billion dollars and increasing. Although many consumers view herbal dietary supplements as a holistic alternative to achieving improved health and weight loss, the supplements have a wide variety of quality and purity with limited proven safety or efficacy that can lead to liver toxicity in susceptible individuals.^9^ Recognition of the potential adverse effects of these therapies is critical for clinicians since identification and cessation of the offending agent is the most important step in management. Case reports regarding specific supplement-related injuries are imperative to delineate the timeline of injuries and to aid in their removal from the marketplace. Albutarex V2’s supplement facts do not show dosages of their “shred complex” and instead list the components of their proprietary blend, making it difficult to discern what component was the likely catalyst to the patient’s presentation (**Table 3**). Although some of the proprietary blend components have been shown to cause liver toxicity in mice, such as piperine and coleus forskolin, there are limited reports for all components of the blend in humans or even in vivo studies.^10,11^ Because of the inability to ascertain what component of the blend caused the DILI, the safest recommendation is to remove the supplement altogether. This case demonstrates the danger of easily accessible “fat burners” and delineates the protracted and prolonged nature of the cholestatic liver injury associated with the particular supplement, Albutarex V2.

## Supplementary Material

usaf175_Supplementary_Data

## Data Availability

The data that support the findings of this study are available on request from the corresponding author. All data are freely accessible.

## INSTITUTIONAL CLEARANCE

Institutional clearance approved.
