# Supplementary material for: Drug-Induced Liver Injury with Novel Fat Burner AlbutarexV2 in an Active Duty Sailor
Source: Mil Med. 2025 May 8;191(1-2):e433–5. doi: 10.1093/milmed/usaf175 (PMC12826854; doi:10.1093/milmed/usaf175)
Supplement: usaf175_Supplementary_Data [file usaf175_supplementary_data.zip › DILI Figure 1 8MAR.pdf]

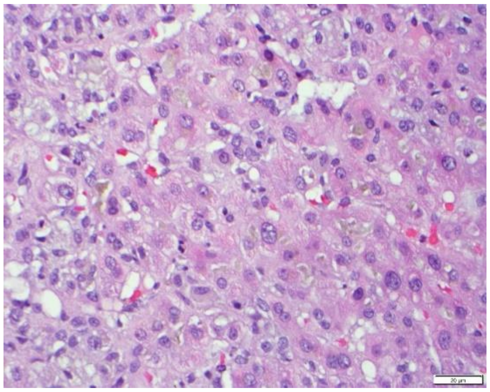

Figure 1: H&E stain of liver biopsy showing inflammation and hepatocellular injury in a perivenular distribution with evidence of significant cholestasis with associated hepatocyte swelling (right)
